# Kuranwendungen mit jodhaltigem Thermalwasser aus thyreologischer Sicht

**DOI:** 10.1007/s10354-020-00782-x

**Published:** 2020-10-07

**Authors:** Peter Mikosch, Eva Trifina-Mikosch, Katharina Saidler, Jennifer Kellner, Susanne Suhrau

**Affiliations:** 1 2. Medizinische Abteilung, Landesklinikum Mistelbach, Mistelbach, Österreich; 2 Medizinische Universität Wien, Externe Lehre, Wien, Österreich

**Keywords:** Schilddrüse, Hyperthyreose, Hypothyreose, Jod, Jodhaltige Heilquellen, Thyroid gland, Hypothyroidism, Hyperthyroidism, Iodine, Spa

## Abstract

Bei einzelnen Schilddrüsenerkrankungen (Immunthyreopathie Morbus Basedow, Immunthyreopathie Hashimoto, Struma mit Autonomie) sollte eine übermäßige Jodzufuhr vermieden werden. Betreffend alimentärer Jodzufuhr gibt es dazu reichliches Informationsmaterial. Kuranwendungen werden zu einem breiten Indikationsspektrum eingesetzt und tragen einen wichtigen Teil zur Therapie gerade chronisch degenerativen Erkrankungen, zur Rehabilitation nach Traumen oder Tumorerkrankungen bei. Eine Jodzufuhr ist auch bei Kuranwendungen mit jodhaltigem Thermalwasser möglich, das Ausmaß der Jodzufuhr ist dabei abhängig von der Applikationsform und der Expositionsdauer. Informationsmaterial dazu ist nur spärlich in der Literatur zu finden. Der Artikel betrachtet jodhaltige Kuranwendungen aus thyreologischer Sicht im Spannungsfeld von positiven und möglichen negativen Auswirkungen einer jodhaltigen Kuranwendung. Neben einem Einblick in die Physiologie und Pathophysiologie des Jodstoffwechsels und Auswirkungen auf einzelne Schilddrüsenerkrankungen wird die Intensität einer Jodaufnahme bei unterschiedlichen Kuranwendungen dargestellt. Eine Übersicht von Kurorten/Thermen mit jodhaltigem Wasser in Österreich und seinen Nachbarstaaten ergänzt von einem Algorithmus zur Selektion von Patienten für eine Kur in Kurorten mit jodhaltigem Wasser wird präsentiert, um mögliche negative jodinduzierte Schilddrüsenfehlfunktionen schon vor Kurantritt zu vermeiden. Dem klinisch tätigen Arzt wird damit eine Unterstützung in der Entscheidungsfindung bei der Auswahl bzw. Ablehnung einer jodhaltigen Kuranwendung für seine Patienten zur Verfügung gestellt.

**Zusatzmaterial online:**

Zusätzliche Informationen sind in der Online-Version dieses Artikels (10.1007/s10354-020-00782-x) enthalten.

## Summary

Excessive iodine intake should be avoided in certain thyroid disorders, such as Graves’ disease, Hashimotoʼs thyroiditis and thyroid goiter with autonomous nodules. Regarding alimentary iodine intake broad information can be found in the literature or the internet. Cure and rehabilitation therapy is used in many clinical settings, such as chronic degenerative disorders of the musculoskeletal system, rehabilitation after trauma, after treatment of malignant tumors and many others. Iodine intake with iodine-rich thermal water during cure and rehabilitation is possible and the extent depends in particular on the route of application and the duration of treatment. Information on this topic can be hardly found in the literature. This article analyses iodine-rich cure and rehabilitation treatment with iodine-rich thermal water from the point of view of the thyroid gland. The positive and possible negative effects of treatment with iodine-rich thermal water on thyroid function are elucidated. Apart from an insight into the physiology and pathophysiology of iodine metabolism, the extent of iodine intake during cure and rehabilitation treatment is presented depending on the route of application and the clinical implications for certain thyroid disorders are discussed. An overview of cure and rehabilitation centers in Austria and neighboring countries is given. In addition, an algorithm for the clinical selection of patients unsuitable for high iodine intake and who should thus avoid an iodine-rich cure or rehabilitation treatment is presented in order to assist clinicians in optimally selecting patients for cure or rehabilitation treatment with iodine-rich thermal water.

Für die Schilddrüsenfunktion stellt Jod einen essenziellen Faktor dar [1]. Zu geringe als auch zu hohe Jodzufuhr kann insbesondere bei Personen mit Schilddrüsenvorerkrankungen pathologische Veränderungen der Schilddrüsenfunktion hervorrufen. Jod kommt in unterschiedlichen chemischen Verbindungen in größeren Mengen nur im Meer (in Meeresalgen mit Konzentration von Jodid bis auf eine 3000-fache Konzentration im Vergleich zu Jodid im Meerwasser) sowie in heißen Quellen aus dem Erdinneren vor [2]. Die Möglichkeiten einer Jodzufuhr im täglichen Leben sind jedoch vielfältig [1]. Neben einer gezielten Jodzufuhr (z. B. jodiertes Speisesalz, Jodierung von Trinkwasser) zwecks Beseitigung von Jodmangel, welcher in vielen Weltregionen vorhanden ist, gibt es auch diverse Jodquellen (Fischprodukte, Algenprodukte, einzelne Mineralwässer, jodhaltige Röntgenkontrastmittel, jodhaltige Medikamente), die zu einer hohen Jodbelastung mit negativen Folgen für Personen mit Schilddrüsenerkrankungen führen können [1, 3, 4].

Als Bestandteil der Schilddrüsenhormone Trijodthyronin und Tetrajodthyronin spielt Jod eine zentrale Rolle in der Physiologie und Pathophysiologie der Schilddrüse. Durch Jodmangel kann eine Schilddrüsenunterfunktion hervorgerufen werden, und über Jahre kann es zur Entwicklung einer diffusen und/oder nodulären Struma durch Hyperplasie der Schilddrüsenfollikel kommen. Gleichzeitig kann sich die Menge autonomer Schilddrüsenzellen in der gesamten Schilddrüse bzw. einzelnen Schilddrüsenknoten vermehren mit der Folge einer diffusen oder nodulären Schilddrüsenautonomie. Werden Patienten, die einem langjährigen Jodmangelzustand ausgesetzt waren, mit größeren Jodmengen exponiert, können Schilddrüsenautonomien dekompensieren und eine Hyperthyreose hervorrufen. Bei Immunthyreopathien (Immunthyreopathie Hashimoto, Morbus Basedow) kann eine hohe Jodexposition ebenfalls eine Hyperthyreose auslösen [1, 2].

Eine tägliche Jodzufuhr von 100–299 µg/l, gemessen als Jodausscheidung im Harn, wird als normal erforderliche Jodzufuhr angesehen [5]. Für erwachsene Personen, die nicht schwanger sind oder stillen, wurde vom US Institute of Medicine in Zusammenarbeit mit der Weltgesundheitsorganisation (WHO), United Nations Children’s Fund (UNICEF) und dem International Council for the Control of Iodine Deficiency Disorders (ICCIDD) eine tägliche Jodzufuhr von 150 µg empfohlen sowie 1100 µg/Tag als tolerable maximale Jodbelastung festgelegt, bei der es bei gesunden Erwachsenen zu keinen negativen Auswirkungen kommen sollte [6, 7]. Das Scientific Committee on Food der Europäischen Union setzte in deren Empfehlung die maximal tolerable Jodmenge für einen normalgewichtigen (60 kg) Erwachsenen mit 600 µg/Tag deutlich niedriger an [8]. Für Österreich, Deutschland und die Schweiz weisen die nationalen Ernährungsgesellschaften darauf hin, dass diese Grenzwerte für bekannte Jodmangelgebiete nicht anwendbar sind und in diesen Gebieten niedriger angesetzt werden müssen [9]. So empfehlen diese Fachgesellschaften, dass die maximale Jodbelastung 500 µg/Tag nicht überschreiten sollte [9].

Österreich stellte bis 1963 ein extremes Jodmangelgebiet mit hoher Prävalenz von Schilddrüsenstruma dar [10]. Gleiches gilt auch für andere Regionen Mitteleuropas abseits von Küstenregionen. In Österreich wurde 1963 eine gesetzlich vorgeschriebene Supplementierung von Speisesalz mit 10 mg Kaliumjodid/kg Speisesalz eingeführt, wodurch die Strumafrequenz verringert [11] und die Jodidausscheidung im Harn bei Kindern verbessert werden konnte [11], nicht jedoch bei Erwachsenen [12]. Durch eine weitere Anhebung der Supplementierung auf 20 mg Kaliumjodid/kg Speisesalz ab 1990 konnte die Jodversorgung der Bevölkerung weiter verbessert bzw. normalisiert werden [13, 14]. Durch die Beseitigung der pathophysiologischen Ursache für die Entstehung der Jodmangelstruma konnte die Strumaprävalenz in Österreich relevant gesenkt werden [10, 14]. Gleichzeitig ergab sich jedoch ein Anstieg der Prävalenz von Immunthyreopathien auf ein Niveau vergleichbar mit Ländern mit guter Jodversorgung [10]. Aus anderen Ländern liegen vergleichbare Ergebnisse zur Minderung des Jodmangels und der Strumaprävalenz vor [15–17], wobei in einzelnen Ländern auch übermäßige Jodzufuhr bzw. fortbestehender Jodmangel festgestellt werden konnte [15–18].

Neben einer Speisesalzjodierung, wie z. B. in Österreich, gibt es auch weitere Möglichkeiten der systematischen Jodzufuhr. Mit Jod versetztes Trinkwasser (Jodierung von Wasser in Jodmangelgebieten oder Jodzugabe zwecks Wasserdesinfektion), Zugabe von Jod im Tierfutter, zu Brot oder anderen Lebensmitteln stellen alternative Möglichkeiten zur Jodzufuhr dar [16–20], um den in vielen Regionen der Welt vorkommenden Jodmangel bzw. wasserhygienische Probleme zu beheben [1].

Kuranwendungen werden mit vielfältigen Indikationen als positiver Beitrag für die Patientengesundheit angeboten [21]. Unter der Vielzahl von Kuranwendungen mit natürlichen Heilwässern werden an einzelnen Kurorten auch jodhaltige Kuranwendungen angeboten, womit diese neben gewünschten positiven Kureffekten auch mögliche negative jodinduzierte Folgen haben können.

Der Artikel betrachtet Kuranwendungen aus thyreologischer Sicht im Spannungsfeld von positiven und möglichen negativen Auswirkungen jodhaltiger Kuranwendungen. Die Betrachtungen sind natürlich auch auf nicht kurmäßige Kontakte mit jodhaltigem Thermalwasser anwendbar, sofern diese wiederholt und über längere Zeiträume erfolgen.

## Physiologie und Pathophysiologie des Jodstoffwechsels bei Jodbelastungen

Organisches und anorganisches Jod wird mit der Nahrung als Jodid im oberen Dünndarm zu über 90 % resorbiert. Von der Schilddrüse wird Jodid aktiv über den Natrium-Jodid-Symporter (NIS) gegen einen Konzentrationsgradienten in die Schilddrüsenzellen aufgenommen. Je geringer der Jodgehalt in der Schilddrüse, desto mehr Jod wird in der Schilddrüse aktiv aufgenommen [2]. Bei ausreichender Sättigung der Schilddrüse mit Jod wird überschüssiges freies Jodid über die Nieren ausgeschieden. Damit ist die Ausscheidung von Jodid über die Nieren ein guter Parameter für die Qualität der Jodversorgung einer Bevölkerung. Hohe Joddosen hemmen direkt die NIS-Aktivität unabhängig von Thyreoidea-stimulierendem Hormon (TSH). Dies wird als Wolff-Chaikoff-Effekt bezeichnet und stellt einen Kompensationsmechanismus der gesunden Schilddrüse zur Prävention einer hyperthyreoten Stoffwechsellage bei hohem Jodplasmaspiegel durch gesteigerte Jodzufuhr (Jodexzess) dar [1, 2]. Die Folge hoher Joddosen ist eine Hypothyreose für die Dauer von etwa 7 bis 14 Tagen. Der Wolff-Chaikoff-Effekt ist selbstlimitierend. Unabhängig von der Jodplasmakonzentration kommt es nachfolgend zur Wiederaufnahme der Hormonsynthese und einer deutlichen Hyperthyreose trotz des erhöhten Jodangebots (Escape-Phänomen) [2].

## Tierstudien mit Jodexposition

Tierstudien, die ein Modell für eine mögliche Exposition mit potenziell hohen Joddosen vergleichbar wie bei einer Kuranwendung darstellen, ergaben Einblicke in zugrunde liegende zelluläre Veränderungen, ausgelöst durch Jodexposition.

Arriagada et al. [22] konnten in einem In-vitro-Model an Rattenzellen zeigen, dass eine Exposition mit hohen Joddosen für 1 bis 2 Tage zu einer Inhibition der Schilddrüsenhormonsynthese über den bekannten Wolff-Chaikoff-Effekt führt. Dies wird über eine gesteigerte H_2_O_2_-Produktion generiert, die nachfolgend zu einer Reduktion der NIS-Expression führt. Die Blockade trat innerhalb von 2–5 h nach Jodexposition auf. Reaktive Sauerstoffderivate konnten diese Reaktion wieder aufheben [22]. Lebsir et al. [23] analysierten die Auswirkungen einer Jodexposition mit 1 mg/kg über die Dauer von 1, 4 und 8 Tagen bei erwachsenen Ratten. Die Schilddrüsenhormone zeigten keine Veränderung im Beobachtungszeitraum, es ergab sich jedoch ein Wolff-Chaikoff-Effekt mit prompter Erniedrigung der NIS und MCT8-mRNA-Expression um −58 % bzw. −26 %. Nachfolgend ergab sich auch eine verminderte Expression von Thyreoperoxidase(TPO)-mRNA um −33 % in Kombination mit einer vermehrten Stimulation von „pendred syndrome gene“(PDS)-mRNA um +62 %. Somit konnte gezeigt werden, dass eine Jodexposition eine prompte Veränderung der Genexpression im Zusammenhang mit der Synthese und Sekretion von Schilddrüsenhormonen bedingt [23].

Im Gegensatz zur kurzfristigen Jodexposition ergaben Studien mit längerfristiger Jodexposition andere Ergebnisse betreffend Schilddrüsenhormonwerte. Joddefiziente Ratten erhielten 20 µg/kg Jodid für die Dauer von 17 Monaten als transdermale Mikroemulsion. Nach 4 Wochen Exposition stiegen die T3- und T4-Werte signifikant an, und TSH fiel signifikant ab, die Jodidausscheidung im Harn erhöhte sich signifikant [24]. Vergleichbare Ergebnisse ergaben sich in einer weiteren Studie durch Applikation einer transdermalen Jodidmikroemulsion bei Sprague-Dawley-Ratten. Innerhalb von 4 Wochen kam es zu einem Anstieg von T3 und T4 und einem Abfall von TSH bei gleichzeitigem Anstieg der Harnjodidausscheidung [24]. Diese Ergebnisse einer längerfristigen Jodexposition sind somit mit einer jodinduzierten Hyperthyreose vereinbar.

## Überdurchschnittliche Jodzufuhr

Bei gesunden Personen wird eine hohe bzw. überdurchschnittliche Jodzufuhr in der Regel gut vertragen [25]. Bei einzelnen Personen kann jedoch eine überdurchschnittliche Jodzufuhr zu gehäuftem Auftreten von latenter/manifester Hypothyreose, Hyperthyreose, einem Anstieg von Schilddrüsenautoantikörpern und zu gehäuftem Auftreten von Immunthyreopathien führen [1, 16, 18, 25]. Gerade Personen, die vorab einen Jodmangelzustand aufwiesen, haben eine erhöhte Gefährdung betreffend Entwicklung einer Schilddrüsenfehlfunktion [25]. Auch bei Patienten mit vorangegangener Post-partum-Thyreoiditis, Morbus Basedow, Immunthyreopathie Hashimoto, Therapie mit Interferon bzw. Amiodaron zeigte sich gehäuft eine gestörte Schilddrüsenfunktion nach Jodexposition [26].

Quellen einer überdurchschnittlichen Jodzufuhr bzw. eines Jodexzesses können neben einer übermäßigen Jodzufuhr über die Nahrung bzw. jodiertem Speisesalz auch Medikamente (Amiodaron), topisch appliziertes Jod als Desinfektionsmittel bzw. Antiseptikum [Polyvidon-Jod – PVP: Poly(1-(2-oxo-1-pyrrolidinyl)ethylen)iod-Komplex], jodhaltige Vaginal- oder Mundspülungen, jodhaltige Zahnpasten, jodhaltige Röntgenkontrastmittel sein [1]. In Einzelfällen kann zu Desinfektionszwecken jodiertes Trinkwasser bei Personen mit Schilddrüsenvorerkrankungen zu einer Hyperthyreose führen [19]. Auch haben einzelne Mineralwässer einen hohen Jodanteil [4], und es wird empfohlen, dass jodhaltige Lebensmittel und Mineralwässer bei Erkrankungen der Schilddrüse, die zur Hyperthyreose neigen können (Struma mit Autonomie, Morbus Basedow), gemieden werden sollten [4].

## Heilquellen

Eine Heilquelle ist ein Heilvorkommen, das aufgrund von besonderen Eigenschaften ohne Abänderung der natürlichen Zusammensetzung eine wissenschaftlich anerkannte Heilwirkung entfalten kann [21]. Heilquellen enthalten typischerweise relevante Anteile an Eisen, Magnesium, Kalzium, Jod, Schwefel, schwach radioaktiven Bestandteilen, wie z. B. Radon, gelöstes Kochsalz (Sole) oder natürliche Kohlensäure, wobei für die Anerkennung als Heilquelle ein Mindestgehalt an gelösten festen Stoffen von 1 g/kg Wasser vorliegen muss. Liegt eine Mineralisierung unter 1 g/kg Wasser vor, wird von akratischen oder mineralarmen Heilquellen gesprochen. Auf der Basis der Austrittstemperatur einer Heilquelle wird auch zwischen kalten Heilquellen mit einer Temperatur unter 20 °C und Thermalquellen mit einer Temperatur über 20 °C unterschieden [21].

Die Nutzung von Thermalquellen hat eine lange Geschichte [27, 28]. Als älteste Reste einer Heilquelle im Alpenraum wurde eine über 3000 Jahre alte Quellfassung aus der Bronzezeit mit keltischem Ursprung bei einer Quelle in St. Moritz gefunden. In römischer Zeit wurden Mineral- und Thermalquellen in allen Teilen des römischen Reiches genutzt und stellten einen wichtigen Bestandteil damaliger Lebenskultur dar. Römische Feldlager wurden oft in der Nähe heißer Quellen angelegt und waren dadurch die Basis vieler heute noch bekannter Thermenorte, wie z. B. Aquae Mattiacae (Wiesbaden), Aquae Gratianae (Aix les Bains), Aquae Sextiae (Aix en Provence), Vicus Aquarum (Baden in der Schweiz), Civitas Aquensis (Baden-Baden), Aquae Sulis (Bath) und Aquisgranum (Aachen). Auf dem Gebiet von Österreich waren die warmen Schwefelquellen von Baden bei Wien und die Thermalquelle von Villach schon zur Römerzeit bekannt. Reste römischer Bäder bzw. Thermen in Deutschland (z. B. Barbarathermen und Kaiserthermen in Trier, Römerbad Jagsthausen, Römerbad in Badenweiler), Österreich (z. B. Carnuntum) und Schweiz (z. B. Baden/Aquae Helveticae) zeugen auch heute noch von dieser frühen Verbreitung einer Bäderkultur nördlich der Alpen bzw. in Mitteleuropa.

## Kuraufenthalte

In Österreich werden Kuraufenthalte in der Regel für 3 bis 4 Wochen gewährt [21, 28]. Kuranwendungen haben eine breite Indikationsstellung insbesondere bei Erkrankungen des Bewegungsapparates, des Herz-Kreislauf-Systems, arterieller Hypertonie, Bronchitis, degenerativen Augenerkrankungen, psychischen Erkrankungen, Erkrankungen des Immunsystems zwecks Regeneration bzw. Stärkung der Organfunktionen und können in unterschiedlichster Form angewendet werden (z. B. Bad, Inhalation, Trinkkur, Packungen, Iontophorese) [25, 28–30].

## Jodhaltige Kuranwendungen

Diverse Elemente wie Jod, Selen, Brom und Eisen spielen eine wichtige Rolle bei der Schilddrüsenhormonproduktion bzw. der Steuerung der Schilddrüsenfunktion [31, 32]. Aus thyreologischer Sicht ist insbesondere die Jodzufuhr eine relevante Frage bei einer Kuranwendung, kann doch Jod bei jodhaltiger Kuranwendung von extern (Bad, Packungen) oder intern (Trinkkur, Inhalation) ggf. in größerer Menge während einer 3‑ bis 4‑wöchigen Kur zugeführt werden [33]. Historisch gesehen wurde schon frühzeitig auf die heilende Wirkung von jodreichen Thermalquellen hingewiesen [24, 33–35]. So z. B. wurde für die stärkste jodhaltige Thermalquelle Österreichs in Hall/Oberösterreich die positive Wirkung des Thermalwassers bei Kropferkrankungen schon 1777 von Heinrich Johann Nepomuk von Crantz im Werk „Gesundbrunnen der österreichischen Monarchie“ lobend erwähnt [36]. Neben einer allgemein vitalisierenden und belebenden Wirkung konnten positive Effekte von jodhaltigen Kuranwendungen auf Herz-Kreislauf-System, rheologische Eigenschaften des Bluts, Schilddrüsenvergrößerung, Lipidmetabolismus, Lungenfunktion und bei Augenerkrankungen beschrieben werden [21, 28, 29, 33]. Auch bei Sekretionsanomalien des Magens und entzündlichen Magen-Darm-Erkrankungen liegen positive Ergebnisse für Jod als Heilmittel vor [35]. Auf der Basis dieser Erkenntnisse stellen aktuell arterieller Hypertonus, Arteriosklerose, chronische Bronchitis, chronische Venenerkrankungen, Jodmangelzustände, entzündliche Magen-Darm-Erkrankungen, Erkrankungen des Stütz- und Bewegungsapparates sowie diverse Haut- und Augenerkrankungen Indikationen für Kuranwendungen mit jodhaltigem Thermalwasser dar [21, 27, 28, 33, 35, 37, 38].

Einzelne Kuranstalten bzw. Kurmittelverzeichnisse erwähnen neben den vielfältigen Indikationen zu den Kuranwendungen auch mögliche Nebenwirkungen einer jodhaltigen Kuranwendung auf die Schilddrüse, wie z. B. temporäre Hypothyreose [29], bzw. medizinische Kontraindikationen von jodhaltigen Kuranwendungen [28, 29, 37–39]. Bei einzelnen Thermen mit jodhaltigen Thermalwässern wird folglich explizit darauf hingewiesen, dass Patienten mit Schilddrüsenvorerkrankungen, insbesondere bekannte Hyperthyreose, bedingt durch Schilddrüsenautonomie oder Immunerkrankungen der Schilddrüse, für eine Kuranwendung mit jodhaltigen Kurmitteln nicht geeignet sind [37–39].

Aus thyreologischer Sicht ergeben sich für die Klinik mehrere Fragen: Können Patienten, die keine Schilddrüsenvorerkrankung haben, aus thyreologischer Sicht ohne Bedenken eine Kuranwendung mit jodhaltigem Thermalwasser durchführen? Welchen Patienten mit einer Schilddrüsenvorerkrankung können keine jodhaltigen Kuranwendungen empfohlen werden, und bei welchen Konstellationen ist ein Kurantritt unter dem Gesichtspunkt einer Risiko-Nutzen-Abwägung möglich? An welchen Kurorten gibt es jodhaltige Kuranwendungen? Nach welchem Algorithmus könnte eine Selektion von Patienten vor Kurantritt vorgenommen werden, um das Risiko möglicher negativer Auswirkungen einer erhöhten Jodzufuhr zu minimieren?

## Jodaufnahme bei Kuranwendungen

Bei Kuranwendungen kann Jod über unterschiedliche Wege aufgenommen werden. Zu unterscheiden sind dabei die unterschiedlichen Applikationsformen von jodhaltigem Wasser. In Bad Hall, einer der stärksten Jod-Brom-Sole-Quellen Mitteleuropas, erfolgten über die Paracelsus-Gesellschaft für Balneologie und Jodforschung ausführliche Untersuchungen zur Jodaufnahme [30, 40]. Messungen von Jod im Sammelharn und bezogen auf die Kreatininausscheidung geben einen guten Hinweis auf die Jodidresorption bei einzelnen Kuranwendungen. Jodverteilungsstudien wurden mithilfe von radioaktiv markiertem Jod durchgeführt. Von 1 mg oral zugeführtem Jodid – dies entspricht etwa der Tagesmenge während einer Trinkkur in Bad Hall – werden 90 % in 24 h wieder ausgeschieden. Die durchschnittliche Ausscheidung von Jod/g Kreatinin (Crea) beträgt ohne Kuranwendung durchschnittlich 130 µg/g Crea. Nach einer Trinkkur mit 100 ml Tassiloquelle, Bad Hall, steigerte sich die Jodausscheidung auf 2450 µg, nach Bädern auf 350 µg, nach Überwärmungspackungen auf 370 µg, nach Ultraschallinhalationen auf 240 µg, nach Augeniontophorese auf 530 µg. Nach dem Trinken steigt das Serumgesamtjod kurzfristig auf einen Wert von 35 µg/100 ml, die Schilddrüsenhormone änderten sich nur geringfügig, das freie anorganische Jodid stieg zwischenzeitig auf das 40-Fache an [30, 40–42].

Vergleichbare Ergebnisse konnten metabolische Untersuchungen in Salsomaggiore, Italien, erbringen [43–45]. Bacolla et al. stellten fest, dass beim Baden Jod vorrangig inhalativ aufgenommen wird und nicht, wie zu vermuten wäre, über die intakte Haut [43]. Jod wird aus dem Badewasser durch das Vorliegen von Hypochlorit freigesetzt und reichert sich knapp über der Wasseroberfläche an. Das freigesetzte Jod wird dann während des Badens eingeatmet [43]. Bacolla et al. [43, 44] untersuchte in deren Studien die während therapeutischer Kuranwendungen aufgenommenen Jodmengen beim Baden, beim Inhalieren und durch Ingestion. Die Harnjodidausscheidung vor dem Baden in jodhaltigem Thermalwasser war bei 20 untersuchten Schulkindern 186 ± 145 µg Jod/g Crea, nach 20 einstündigen Bädern betrug die Harnjodidausscheidung 386,2 ± 190 µg Jod/g Crea. Bei Inhalationen wurde zwischen „trockenen“ und „feuchten“ Inhalationen unterschieden. In Testserien nach 10 trockenen Inhalationen (Inhalation mit 398 ± 37 µg Jod/m^3^) wurde errechnet, dass 151,6 µg Jod pro Inhalation aufgenommen wurde. Bei einer zweiten Testserie mit feuchten Inhalationen (Inhalation mit 287 ± 90 µg Jod/m^3^) ergab sich eine mittlere Inhalation von 109,3 µg Jod pro Applikation. Die Harnjodidausscheidung stieg um 82 ± 23 µg Jod/g Crea bis zum Ende der Applikationen an [43]; 24 h nach jodhaltigen Inhalationen fanden sich 34 %, nach Ingestion von jodhaltigem Wasser 87 % der zugeführten Jodmenge im Körper [43]. Die zugeführten Jodmengen wurden nachfolgend rasch über die Nieren ausgeschieden, und die größten Jodmengen konnten in den ersten Urinproben nach Exposition festgesellt werden [43]. In deren metabolischer Studie kommen die Autoren zum Schluss, dass die aufgenommenen Jodmengen zu keinem Zeitpunkt Konzentrationen erreichen, die bei einer gesunden Schilddrüse zu einer Änderung der Schilddrüsenfunktion führen könnten [43]. Nach Trinken von jodhaltigem Wasser über 8 Tage hinweg konnten die Autoren eine Erhöhung der Harnjodidausscheidung von initial 100 µg Jod/24 h Harn auf 244µg Jod/24 h Harn bzw. eine Aufnahme von 17 % der zugeführten Jodmenge feststellen [44]. In einer weiteren Analyse ergab sich nach Trinken von radioaktiv markiertem Jod eine Retention von 13 % der zugeführten Jodmenge im Körper innerhalb von 24 h [44].

Fälle von Änderungen der Schilddrüsenfunktion nach Jodexposition bei Kuren sind in der Literatur nur selten dokumentiert. Ein Fall einer jodinduzierten Hypothyreose durch exzessive transkutane/transmuköse Absorption von Jod wurde nach einer 3‑monatigen Kur mit jodiertem Badesalz berichtet [46].

## Orte bzw. Kuranstalten mit jodhaltigen Quellen

Zum vorliegenden Artikel wurde vom Autorenteam eine umfassende Recherche zu jodhaltigen Heilvorkommen in Österreich und seinen Nachbarstaaten durchgeführt. Dabei wurden verfügbare Informationen aus dem Internet bzw. Literatur zu „jodhaltiges Wasser“, „jodhaltige Kuranwendungen“, „Jod und Heilvorkommen“ und „Jod und Kuranwendungen“ erhoben. Die angeführten Informationen beziehen sich auf den Informationsstand Frühjahr 2020. Eine gezielte Zugabe von Jod bei Kuranwendungen ist prinzipiell möglich und wird in einzelnen Kurorten, die kein jodhaltiges Thermalwasser aufweisen, auch vereinzelt durchgeführt. Dazu erfolgte keine systematische Recherche.

**Figure 1:**
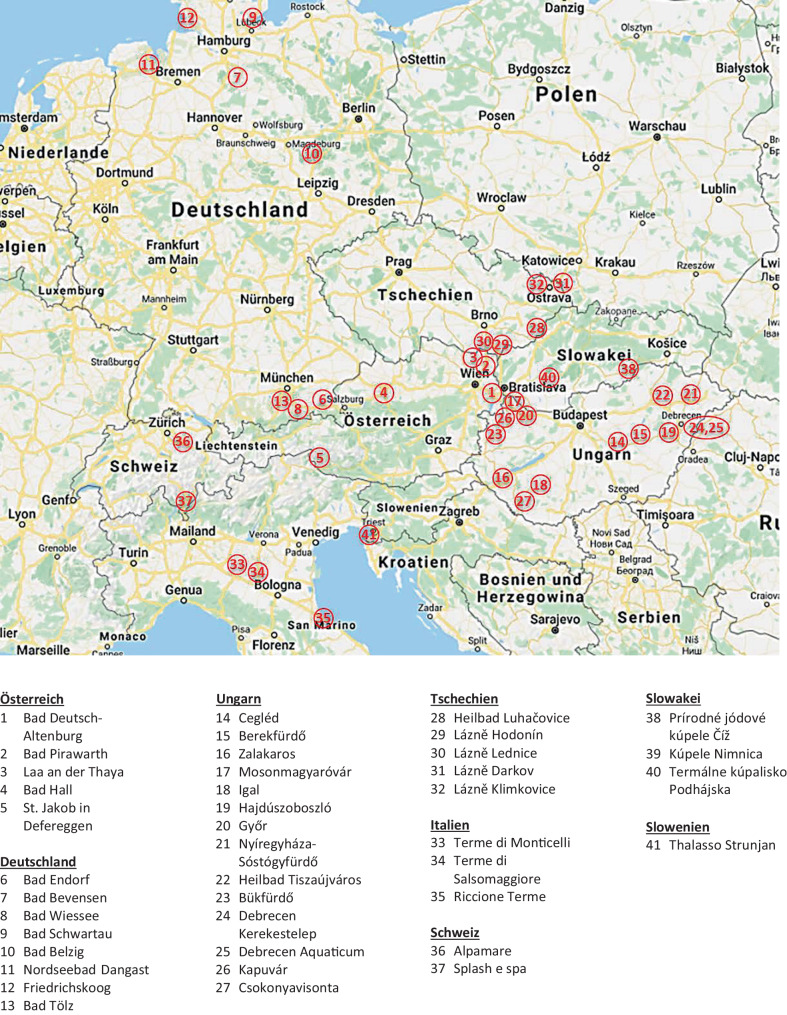

Die Abb. 1 gibt eine geografische Übersicht von den verfügbaren natürlichen Heilvorkommen mit einem Jodgehalt von ≥1 mg Jod/l bzw. kg. Geografische Schwerpunkte mit jodhaltigen Heilvorkommen stehen in engem Zusammenhang mit den geologischen Gegebenheiten der einzelnen Regionen. Die Karte zeigt Schwerpunkte von Thermalorten mit jodhaltigem Wasser in Südbayern, im Dreiländereck Österreich-Tschechien-Slowakei und Österreich-Slowakei-Ungarn, in Süd- und Ostungarn sowie in Italien in der Poebene (Abb. 1). In Tab. 1 sind diese natürlichen Heilvorkommen nach Ländern gegliedert angeführt und die an den Standorten verwendeten Kuranwendungen aufgelistet. Nicht angeführt sind in dieser Tabelle Heilvorkommen mit einem geringeren Jodgehalt als 1 mg/l bzw. kg, Heilvorkommen, die aktuell nicht oder nur für andere Zwecke genutzt werden (z. B. Herstellung von Mineralwasser/Heilwasser) bzw. nicht für Kuranwendungen zur Verfügung stehen. Eine Gesamtübersicht aller natürlichen jodhaltigen Heilquellen von Österreich und den angrenzenden Nachbarstaaten steht als Addendum (Attachment 1) in der Online-Version des Artikels (10.1067/s10354-020-00782-x) zur Verfügung.

**Table 1:** 

Ort	Karte	Jod mg/l	Jod mg/kg	Literatur	Anwendungen
**Österreich**					
Bad Deutsch-Altenburg: Kurzentrum Ludwigstorff	1	1,05–1,3	–	GBA Wien 2018	Bäder, Inhalation
Bad Pirawarth: Klinik Pirawarth	2	12,2	–	GBA Wien 2018	Solebäder, Jodidbäder
Bad Hall: Eurothermenresort	3	26,3–44,5	–	GBA Wien 2018	Jodsolebad, Inhalation, Trinkkur, Packungen, Augenbehandlungen
Laa an der Thaya: Therme Laa	4	9,05	–	GBA Wien 2018	Therme
St. Jakob in Defereggen	5	2,1	–	GBA Wien 2018	Deferegger Heilwasser (seit 2011) für Bäder, Inhalationen, Sprühanwendungen, Wickel
**Deutschland**					
Bad Endorf: Chiemgau-Thermen	6	5	–	HWA TU München	Therme
Bad Bevensen: Jod-Sole-Therme	7	3,4–7,6	–	HWA Institut Fresenius	Bäder, Inhalation
Bad Wiessee: Gesundheitszentrum Jod-Schwefelbad GmbH	8	–	34,6–34,8	Bücher	Bäder, Inhalation
Bad Schwartau: Holstein Therme	9	6,36	–	Website	Therme
Bad Belzig: Stein Therme	10	1,45	–	LU Bad Elster 2015	Bäder
Nordseebad Dangast	11	k. A.	–	Website	Bäder, Inhalation
Friedrichskoog	12	k. A.	–	Website	Heilklima
Bad Tölz	13	k. A.	–	Website	Heilklima, Jodiontophorese, Jodlaugenbäder, Jodseifenabreibungen, Inhalationen
**Ungarn**					
Cegléd: Ceglédi Gyógyfürdő	14	1	–	Website	Bäder
Berekfürdő: Termál Hotel Pávai	15	2,04	–	Website	Bäder
Zalakaros: Heilbad Gránit	16	5,4	–	Website	Bäder
Mosonmagyaróvár: Thermal Hotel	17	1,93	–	Website	Bäder, Trinkkur, Schlamm
Igal	18	3,3	–	Website	Bäder, Trinkkur
Hajdúszoboszló	19	5,5	–	Website	Bäder, Schlamm
Győr: Rába Quelle Bad	20	1,26	–	Website	Bäder, Trinkkur
Nyíregyháza-Sóstógyógyfürdő: Aquarius Erlebnisbad, Parkbad, Freibad am See, Júlia Bad, Hotel Badehaus	21	1,1	–	Website	Bäder
Heilbad Tiszaújváros	22	1,2	–	Website	Bäder, Inhalation, Schlamm
Bükfürdő: Thermal & Spa/Heilbad Bük	23	1,36	–	Website	Bäder, Trinkkur
Debrecen: Kerekestelep	24	1,8	–	Website	Thermalbad
Debrecen: Aquaticum	25	2	–	Website	Bäder
Kapuvár	26	2,52	–	Website	Thermalbad
Csokonyavisonta	27	1,6	–	Website	Heilbad
**Tschechien**					
Heilbad Luhačovice	28	6,9–7,2	–	ZÚ Ostrava 2018	Bäder, Trinkkur, Inhalation
Lázně Hodonín	29	47–56	–	Website	*Info: aufgrund des hohen Jodgehalts wird das Wasser 1:1 mit warmen Wasser verdünnt* Bäder, Inhalation
Lázně Lednice	30	>30	–	Website	Bäder, Inhalation
Lázně Darkov	31	25–45	–	Website	Bäder, Wickel
Lázně Klimkovice	32	40–50	–	Website	Bäder, Wickel
**Italien**^**a**^					
Terme Stufe di Nerone – Bacoili (bei Neapel)	~	3	–	Website	Bäder, Inhalation, Schlamm
Terme di Monticelli	33	40,8	–	Website	Bäder, Inhalation, Schlamm
Terme di Salsomaggiore	34	54–61	–	Website	*„Acqua madre“: konzentriertes Thermalwasser von 1000* *l Brom-Jod-Wasser auf 50* *l Acqua madre* Bäder, Inhalation, Schlamm, orale Balneotherapie
Riccione Terme	35	1,3–7,2	–	Website	Bäder, Inhalation, Schlamm
**Schweiz**					
Alpamare	36	k. A.	–	Website	Jod-Sole-Becken
Splash e spa	37	k. A.	–	Website	Jod-Sole-Becken
**Slowakei**					
Prírodné jódové kúpele Číž	38	k. A.–26,9	–	Website	Bäder, Trinkkur
Kúpele Nimnica	39	1,33	–	Website	Bäder, Inhalation
Termálne kúpalisko Podhájska	40	3,23	–	Website	Thermalbad
**Slowenien**					
Thalasso Strunjan	41	<50	–	Website	Therme mit Meerwasser

^a^Weitere Thermen (ohne Angaben zum Jodgehalt) s. Attachment

## Algorithmus für Selektion von gefährdeten Patienten bei jodhaltigen Kuranwendungen

Ein Kuraufenthalt hat das Ziel, positive gesundheitliche Wirkungen beim Patienten zu entfalten. Dies ist bei Anwendung von jodhaltigen Heilwässern bei einer Vielzahl von Indikationen gegeben (s. oben). Unerwünschte Nebenwirkungen mit Hypothyreose oder Hyperthyreose, wie durch jodhaltige Thermalwässer potenziell auslösbar, sollten, wenn möglich, vermieden werden. Patienten mit einer gesunden Schilddrüse vertragen auch hohe Jodbelastungen gut und haben diesbezüglich keine negativen Auswirkungen auf die Schilddrüsenfunktion zu erwarten [25, 30, 40, 43, 44]. Aus klinischer Sicht wäre es jedoch sinnvoll, Patienten, die eine Schilddrüsenvorerkrankung haben und bezüglich einer Schilddrüsenfehlfunktion potenziell gefährdet sind, schon vor einem Kurantritt durch einen Algorithmus zu erkennen.

**Figure 2:**
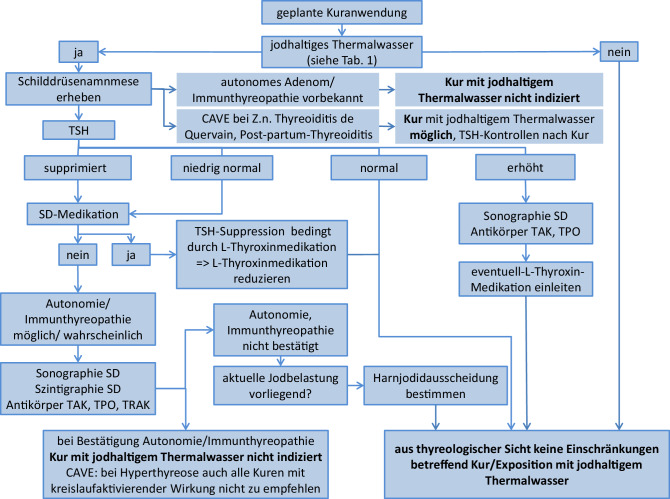

Die erste entscheidende Frage aus Sicht der Thyreologie ist, ob eine Kuranwendung an einem Ort mit jodhaltigen Heilwässern vorgesehen ist (Abb. 1; Tab. 1). Sofern dies der Fall ist, sollten eine gezielte Anamnese betreffend Vorerkrankungen der Schilddrüse sowie eine Laborbestimmung von TSH erfolgen (Abb. 2).

Mögliche relevante Vorerkrankungen wie Schilddrüsenautonomie oder eine Immunthyreopathie, bei denen eine Kuranwendung mit jodhaltigem Thermalwasser gemieden werden sollte, sind gezielt zu erfragen. Diese Erkrankungen sind differenzialdiagnostisch gegenüber anderen Einflussfaktoren, die ebenfalls zu einer TSH-Erniedrigung führen können, wie eine L‑Thyroxin-Medikation oder eine aktuelle Jodbelastung, abzugrenzen (Abb. 2).

Bei Patienten mit bekannten Schilddrüsenerkrankungen, die mit einer Schilddrüsenfehlfunktion einhergehen können (autonomes Adenom, Immunthyreopathie Basedow, Immunthyreopathie Hashimoto), ist die Verordnung von jodhaltigen Kuranwendungen kontraindiziert bzw. große Vorsicht geboten, da durch die Jodzufuhr eine Hyperthyreose ausgelöst werden kann (Abb. 2). Auch Patienten mit abgelaufenen Schilddrüsenentzündungen zeigten nach Jodexposition eine erhöhte Disposition für Schilddrüsenfehlfunktionen, insbesondere Hypothyreose [26, 47]. Diese Patienten können jedoch prinzipiell eine Kur mit jodhaltigen Thermalwässern erhalten, nach der Kur sind jedoch regelmäßige Kontrollen von TSH zu empfehlen, um eine Schilddrüsenfehlfunktion frühzeitig zu erkennen ([47]; Abb. 2).

Eine diagnostische Herausforderung stellen klinisch kompensierte Schilddrüsenerkrankungen dar. Diese fallen ggf. lediglich durch erniedrigte oder niedrig normale TSH-Werte auf. Dies gilt insbesondere in Jodmangelsituationen, bei denen eine bestehende Schilddrüsenautonomie bzw. Immunthyreopathie diese subtile Laborabweichung mit niedrig normalen TSH-Werten hervorrufen kann. Bei hoher Jodbelastung während der Kuranwendung könnte in diesen Fällen aufgrund des vorbestehenden Jodmangels diese kompensierte Schilddrüsenerkrankung durch die Jodzufuhr in eine manifeste Schilddrüsenfehlfunktion dekompensieren. Aus thyreologischer Sicht sollte daher bei Auffälligkeiten in der Schilddrüsenanamnese oder bei erniedrigten bzw. bei niedrig normalen TSH-Werten eine weiterführende Schilddrüsenabklärung vor Kurantritt eingeleitet werden, ggf. in einem Schilddrüsenzentrum, um eine bekannte bzw. klinisch nicht manifeste Schilddrüsenerkrankung näher abzuklären (Abb. 2).

Ob im Einzelfall eine Jodexposition bei vorbekannter Schilddrüsenerkrankung vertretbar ist, sind neben der bekannten Vorerkrankung der Schilddrüse auch die Art und Dauer der Kuranwendungen mit jodhaltigem Thermalwasser sowie der Jodgehalt des Thermalwassers (Tab. 1) mit in eine differenzierte Empfehlung, die den Nutzen einer Kur mit jodhaltigem Thermalwasser gegen die möglichen Risiken einer dadurch ausgelösten Schilddrüsenfehlfunktion gegeneinander abwägt, einzubeziehen.

## Conclusio

Patienten mit einer gesunden Schilddrüse können ohne Bedenken eine Kuranwendung mit hohen Dosen von jodhaltigem Thermalwasser durchführen [25, 30, 40, 43, 44]. Eine signifikante und v. a. anhaltende Änderung der Schilddrüsenfunktion konnte bei verschiedenen Untersuchungen in Tierversuchen als auch bei Kuranwendungen mit unterschiedlichen Zufuhrwegen nicht festgestellt werden [22, 23, 30, 40, 43, 44].

Bei Patienten mit bekannter Schilddrüsenvorerkrankung bzw. Auffälligkeiten von TSH ist eine Kuranwendung mit jodhaltigem Thermalwasser nicht indiziert bzw. erst nach einer thyreologischen Abklärung im Sinne einer Risiko-Nutzen-Abwägung zu verordnen. Eine thyreologische Abklärung sollte in diesen Fällen vor einem Kurantritt in Zusammenarbeit mit einem Schilddrüsenzentrum erfolgen. Neben der bestehenden Schilddrüsenerkrankung sind die Art der Kuranwendung sowie der Jodgehalt der Thermalquelle dabei weitere wichtige Entscheidungsgrößen. All diese Faktoren gilt es, bei einer Risikoabwägung betreffend Kuranwendung mit jodhaltigen Thermalwassern und einer durch die Jodzufuhr ausgelösten Schilddrüsenfehlfunktion zu berücksichtigen.

## Caption Electronic Supplementary Material


