# Supplementary material for: Iodine-rich thermal water in cure and rehabilitation from the perspective of the thyroid gland
Source: Wien Med Wochenschr. 2020 Oct 7;170(15):392–402. [Article in German] doi: 10.1007/s10354-020-00782-x (PMC7593323; doi:10.1007/s10354-020-00782-x)
Supplement: Supplementary file 1 [file 10354_2020_782_MOESM1_ESM.docx]

| **Ort**  Attachment 1 | **Quellen/Bohrungen** | **Wesentliche Bestandteile** | **Jod mg/l** | **Jod mg/kg** | **Literatur** | **Anwendungen/Anmerkungen** |
| --- | --- | --- | --- | --- | --- | --- |

| **Österreich** |  |  |  |  |  |  |
| --- | --- | --- | --- | --- | --- | --- |
| **Bad Deutsch-Altenburg** | Schlossquelle/-brunnen | Na-Ca-Cl-HCO_3_-I-S-Mineral-Thermal-Wasser | 1,05 |  | GBA Wien 2018^(1)^  ÖHKV ^(2)^ | dient als Hauptversorgungsquelle |
|  | Kaiserbadquelle |  | k. A. | k. A. |  |  |
|  | Direktionsquelle/-brunnen |  | 1,3 |  |  |  |
|  | Kurhausbrunnen |  | k. A. | k. A. |  | derzeit nicht in Verwendung |
| Kurzentrum Ludwigstorff |  | I-S-Thermal-Wasser | k. A. | k. A. |  |  |
| **Bad Pirawarth**  (Klinik Pirawarth) | Sophienquelle | I-Sole | 1998: 12,2 |  | GBA Wien 2018 ^(1)^ | Solebäder, Jodidbäder |
|  | Parkquelle | Ca-Mg-S-HCO_3_-Mineralwasser | / |  | ÖHKV ^(2)^ | Trinkwasser – frei zugänglich |
| **Bad Hall**  (Eurothermenresort)  (Angabe der Quellen mit aufrechtem Wasserrecht zur Nutzung durch die Tassilo Kurbetriebe) | Tassiloquelle | I-Sole | 26,3 |  | GBA Wien 2018 ^(1)^ | Jodsole-Bad, Inhalation, Trinkkur, Packungen, Augenbehandlungen |
|  | Paracelsusquelle |  | 44,5 |  |  |  |
|  | Sulzbachquelle II |  | 31,4 |  |  |  |
|  | Zehrmühle |  | 34 |  |  |  |
|  | Guntherhöhenquelle |  | 39 |  |  |  |
|  | Johannesquelle |  | / |  |  |  |
|  | Holznerquelle |  | / |  |  |  |
|  | Feyreggerbach II |  | / |  |  |  |
|  | Sonde V18 |  | 40,6 |  |  |  |
|  | Feyreggerbach III |  | 42,8 |  |  |  |
| **Bad Goisern** | Schwefelquelle | akratische Na-Cl-HCO_3_-S-Quelle, titierbarer Schwefel 2,4 mg/l | 0,25 |  | GBA Wien 2018 ^(1)^ | Kurbetrieb in diversen Hotels privat möglich |
| **Laa an der Thaya** | Thermal Nord 1 | Na-Cl-I-Mineral-Thermalquelle | 9,05 |  | GBA Wien 2018 ^(1)^ | Therme |
|  | Vitus-Brunnen I |  | 0,15 |  |  | Anerkennung als Mineralwasservorkommen |
|  | Vitus-Brunnen II |  | / |  |  |  |
|  | Thermal Süd 1 (Egerium) |  | 66 |  |  | verschlossen |
|  | Thermal Süd 1 |  | 18,55 |  |  |  |
| **Engelhartstätten** | Wiestalquelle | Na-Cl-S-I-Mineral-Thermal-Wasser | k. A. | k. A. | GBA Wien 2018 ^(1)^ | 05.02.2001 Zurücknahme der Anerkennung der Wiestal-Heilquelle als Heilquelle (Salzburger Landesregierung) |
| **Neumarkt/Ybbs** | zwei Tiefbrunnen | Na-Cl-HCO_3_-I-Mineral-Wasser  Na-Cl-I-Mineral-Wasser | k. A. | k. A. | GBA Wien 2018 ^(1)^ | ehemalige Heilquelle |
| **Reichersberg** | Tiefbohrung Reichersberg 1  Therme Johannesbad Bad Füssing | S-Na-HCO_3_-Cl-Thermalwasser | k. A. | k. A. | GBA Wien 2018 ^(1)^  Inst. f. Wasserchemie und chem. Balneologie der TU München | Nutzung für Therapie- und Kurbetriebe |
|  | Tiefbohrung Reichersberg 2 |  | 0,48 |  |  | Nutzung als Beobachtungsbrunnen |
| **Speltenbach** |  | Fe-haltige Na-Cl-I-Thermal-Sole | 108 |  | Internet, keine offiziellen Angaben | Speltenbacher Thermalwasser GmbH 🡺 nicht auffindbar/Produktion eingestellt? |
| **St. Kanzian** | Thermal 1 | I-haltige Na-Cl-HCO_3_-Thermal-Sole | 1,27 |  | GBA Wien 2018 ^(1)^ | Bohrloch derzeit verschlossen |
| **Loipersdorf bei Fürstenfeld** | Grieselstein Thermal 1 | Na-Cl-HCO_3_-Thermal-Wasser | 2008: 0,1 |  | GBA Wien 2018 ^(1)^ | Therme – nur privat möglich |
|  | Lautenberg 1 |  | 1996: 0,387 |  |  |  |
|  | Binderberg 1 |  | 1996: 0,216 |  |  |  |
| **Sicheldorfer Heilwasser** | Josefsquelle | Na-Cl-HCO_3_-Heilwasser  I-haltiger Na-HCO_3_-Säuerling | 1990: 0,91 |  | GBA Wien 2018 ^(1)^ | Heilwasser |
| **Fürstenfeld (Stmk)** | Thermal 1 | Na-Cl-Typ  Formationswasser mit erhöhten Konzentrationen von Fe und Jodid | 8 | das Salz (1 kg/24 l Thermalwasser) enthält 108 mg/kg | GBA Wien 2018 ^(1)^ | seit 1997 Förderung geringer Menge hochmineralisierten Thermalwassers zur Produktion von Heil- und Badesalz |
| **Leopoldskron (Sbg)** | Obermoos Thermal 1 |  | 0,95 |  | GBA Wien 2018 ^(1)^ | wird bis heute nicht genutzt |
| **St. Jakob in Defereggen (Tirol)** | Thermal 1 | Br-I-Sole | 2,1 |  | GBA Wien 2018 ^(1)^ | Deferegger Heilwasser für Bäder, Inhalationen, Sprühanwendungen, Wickel (Abfüllung seit 2011) |

1. Elster D., Fischer L., Hann S, Goldbrunner J, Schubert G., Berka R., Hobiger G., Legerer P., Philippitsch R. Österreichs Mineral- und Heilwässer. Geologische Bundesanstalt Wien 2018 im Auftrag des Bundesministeriums für Nachhaltigkeit und Tourismus
2. Österreichischer Heilbäder- und Kurorteverband

| **Deutschland** |  |  |  |  |  |  |
| --- | --- | --- | --- | --- | --- | --- |
| **Bad Griesbach**  Wohlfühl-Therme | Nikolaus-Quelle | F-Na-HCO_3_-Cl-Thermalwasser |  | 0,6 | Heilwasseranalyse:  TU München | Therme |
|  | Marien-Quelle |  |  | 0,62 |  |  |
|  | Karls-Quelle |  |  | 0,53 |  |  |
| **Bad Birnbach**  Rottal-Therme | Chrysantiquelle | F-Na-HCO_3_-Cl-Thermalwasser | 2018: 0,5 |  | Heilwasseranalyse:  UIS Umweltinstituts synlab GmbH | Therme |
|  | Konradsquelle |  | k. A. | k. A. |  |  |
| **Bad Endorf**  Chiemgau-Thermen | Bohrung 2 | I-F-Na-Cl-Thermalwasser | 1973/94: 47,2 |  | Heilwasseranalyse:  TU München | Therme  Fast alle Becken werden durch die Bohrung 3 gespeist – außer das Sommer-Aktiv- u. Trainingsbecken im EG. |
|  | Bohrung 3 |  | 5 |  |  |  |
| **Bad Bevensen**  Jod-Sole-Therme | Bohrung 1 | I-haltige Sole | 1975: 3,6  2011: 3,4 |  | k. A. | Bäder, Inhalation |
|  | Bohrung 2 | I-haltige Thermal-Sole | 1987: 7,6  2008: 7,1 |  | Heilwasseranalyse: Institut Fresenius |  |
| **Bad Wiessee**  Gesundheitszentrum Jod-Schwefelbad GmbH | Wilhelmina-Quelle | I-S-NaCl-Quelle |  | 34,8 | Bücher (*)(**)(***) | Bäder, Inhalation |
|  | Adrianus-Quelle (König Ludwig III-Quelle?) |  |  | 34,6 |  |  |
| **Bad Wörishofen**  Therme Bad Wörishofen | k. A. | I-F-Wasser | 2009: 0.89 |  | Heilwasseranalyse:  TU München | Therme, I-Se-Bad = angereicherter Bade-zusatz (meist 2-3%ige Jod-Selen-Lösung) |
| **Bad Schwartau**  Holstein Therme | Anton-Baumann-Quelle | I-Sole | 6,36 |  | https://holstein-therme.de/gesundes-nass.php | Therme |
| **St. Leonhards Quellen**  Tafel- und Mineralwasser |  |  | 37 µg/100 ml Tafelwasser |  | St. Leonhards-Vertriebs GmbH&Co. KG | Jod Natur Tafelwasser (Tafelwasser wird mit jodhaltiger Urmeersole im Verhältnis 54:1 angereichert) |
| **Bad Belzig**  Stein Therme | k. A. | I-Sole | 2015: 1,45 |  | Laborunion Bad Elster 01.04.2015 | Bäder |
| **Nordseebad Dangast** | Dangaster Quelle (?) | I-Sole  Nordseebad: 80% Süßwasser und 20% Jod-Sole aus der Quelle | k.A. | k. A. | https://www.rehakliniken.de/rehakliniken/friesenhoern-nordsee-kliniken-dangast | Bäder, Inhalation |
| **Friedrichskoog** |  |  | k.A. | k. A. | https://www.rehakliniken.de/kurorte/friedrichskoog | Heilklima – bei Ebbe höhere Jod-konzentrationen in der Luft nachweisbar |
| **Bad Tölz** |  |  | k.A. | k. A. | https://www.bad-toelz.de/de/gesundheit/urlaub-im-heilklima.html | Heilklima, Jod-Iontophorese, Jod-laugenbäder, Jodseifen-Abreibungen, Inhalationen mit J-Na-Cl-Wässern |

(*) Balneologie und medizinische Klimatologie; Band 2: Balneologie; Springer-Verlag; A. Amelung; G. Hildebrandt; ISBN-13: 978-3-642-70131-3

(**) Lehrbuch der Bäder- und Klimaheilkunde 1. Teil; H. Vogt; W. Amelung; A. Bacmeister; Springer-Verlag 1940

(***) Wasser – Untersuchung, Beurteilung, Aufbereitung, Chemie, Bakteriologie, Biologie; Dr. phil. Karl Höll; Walter de Gruyter&Co 1970

| **Ungarn** |  |  |  |  |  |  |
| --- | --- | --- | --- | --- | --- | --- |
| **Cegléd**  Ceglédi Gyógyfürdő |  |  | 1 |  | http://cegledfurdo.hu/rolunk/ | Bäder |
| **Berekfürdő**  Termál Hotel Pávai |  |  | 2,04 |  | http://www.termalhotelpavai.hu/gyogyaszat.html | Bäder |
| **Zalakaros**  Heilbad Gránit |  |  | 5,4 |  | https://hellozalakaros.hu/de/was-empfehlen-wir | Bäder |
| **Mosonmagyaróvár**  Thermal Hotel |  |  | 1,93 |  | http://www.thermal-movar.hu/ger/thermalwasser.html | Bäder, Trinkkur, Schlamm |
| **Igal** |  |  | 3,3 |  | http://www.igal.hu/Gyogyfurdo/Az-Igali-gyogyviz | Bäder, Trinkkur |
| **Hajdúszoboszló** |  |  | 5,5 |  | https://hungarospa.hu/de/Heilbad/Heilwasser | Bäder, Schlamm |
| **Győr**  Rába Quelle Bad |  |  | 1,26 |  | https://www.rabaquelle.hu/de/das-bad/das-heilwasser/ | Bäder, Trinkkur |
| **Nyíregyháza-Sóstógyógyfürdő**  Aquarius Erlebnisbad, Parkbad, Freibad am See, Júlia Bad, Hotel Badehaus |  |  | 1,1 |  | https://www.aquariusspa.hu/hu/gyogyvizunkrol | Bäder |
| **Heilbad Tiszaújváros** |  |  | 1,2 |  | http://www.termal.tujvaros.hu/index.php/en/water-quality | Bäder, Inhalation, Schlamm |
| **Bükfürdő**  Thermal & Spa/Heilbad Bük |  |  | 1,36 |  | https://www.bukfurdo.hu/de/heilwasser | Bäder, Trinkkur |
| **Debrecen**  Kerekestelep |  |  | 1,8 |  | http://kerekestelepifurdo.hu/a-gyogyviz/ | Thermalbad |
| **Debrecen**  Aquaticum |  |  | 2 |  | https://spa.aquaticum.hu/de/heilwasser | Bäder |
| **Kapuvár** |  |  | 2,52 |  | http://www.kapuvar.hu/varos/termeszet/ | Thermalbad |
| **Csokonyavisonta** |  |  | 1,6 |  | http://www.termaludulo.hu/leistungen.html | Heilbad |
| **Heilbad Harkany** |  |  | 0,12 |  | https://www.harkany-hotel.de/heilwasser-von-harkany/ | Bäder, Trinkkuren |
| **Heilbad Komáron** |  | Ca-Mg-HCO_3_-Cl-Heilwasser | 0,082 |  | http://www.komthermal.hu/dienstleistungen/therapien | Bäder, Trinkkuren |
| **Heilbad Gyopáros** | Heilbrunnen Gyopáros | Alkalisches HCO_3_-Heilwasser | 0,09 |  | https://gyoparosfurdo-oroshaza.hu/gyogyaszat/gyogyvizunk |  |
| **Heilbad Csongrád** |  | F-Wasser | 0,04 |  | https://csongradfurdo.hu/vizosszetetel/ |  |
| **Heilbad Kehidakustány** |  |  | 0,028 |  | https://kehidatermal.hu/de/gyogy-es-elmenyfurdo/gyogyfurdo |  |
| **Heilbad Lenti** |  |  | 0,01 |  | http://lentifurdo.hu/de/heilkraft/heilbad/zusammensetzung-des-heilwasser/ |  |
| **Heilbad Cserkeszölö**  Medizinisches Zentrum |  |  | 0,61 |  | http://www.cserkeszolofurdo.hu/de/medizinisches-zentrum | Thermalbad |
| **Heilbad Balf** |  | S-haltig – keine I-Angaben zu finden | k. A. | k. A. |  |  |
| **Heilbad Sárvár** |  |  | 0,32 |  | www.savarfurdo.hu |  |
| **Bad Héviz** |  |  | 0,021 |  | https://www.heviz.hu/hu/hevizi-to/hevizi-tofurdo/hevizi-to-viz-osszetetele | Thermalsee |
| **Heilbad Agárd** |  |  | 0,074 |  | www.agarditermal.hu | Spa- und Thermalbad |
| **Heilbad Erzsébet** | B-13 |  | 0,13 |  | https://www.elixirhotel.hu/de/medical-wellness/heilbehandlungen |  |
|  | B-40 |  | 0,2 |  |  |  |
| **Heilbad Nagyatád** |  | Na-HCO_3_-Mineralwasser | 0,02 |  | nagyatad.hu/modules.php?name=nagyatad&page=38 |  |
| **Heilbad Árpád** | Innenpools (Belső medencék) |  | 0,46 |  | http://www.arpadfurdo.hu/site/rolunk/ |  |
|  | Außenpools (Kültéri medencék) |  | 0,1 |  |  |  |
| **Burgbad Gyula** | Thermalbrunnen Nr. 1 |  | 0,4 |  | https://varfurdo.hu/de/medizinische-informationen-m226 |  |
| **Hegykő**  Thermalbad Sara |  |  | 0,8 |  | www.saratermal.hu/gyogyviz.php | Thermalbad |
| **Celldőmőlk**  Volcano Spa und Abenteuerbad |  |  | 0,66 |  | https://vulkanfurdo.hu/ | Thermalbad |
| **Budapest**  Gellért Gyógyfürdő |  | Ca-Mg-HCO_3_-Na-SO_4_-Cl-Wasser | 0,7 |  | http://www.gellertfurdo.hu/viz-osszetetele | Thermalbad |
| **Tamási**  Thermal Spa Tamási |  |  | 0,8 |  | https://tamasifurdo.com/de/bad | Thermalbad |

| **Tschechien** |  |  |  |  |  |  |
| --- | --- | --- | --- | --- | --- | --- |
| **Heilbad Luhačovice** | Vincentka (Vinzenzquelle) |  | 7 |  | ZÚ Ostrava 2018  https://www.lazneluhacovice.cz/de/25430-naturliche-heilquellen | Bäder, Trinkkur, Inhalation |
|  | Aloiska (Aloisquelle) |  | 6,9 |  |  |  |
|  | Ottovka (Ottoquelle) |  | 7,2 |  |  |  |
|  | Pramen Dr. Štastného  (Dr. Štastný-Quelle) |  | 9,9 |  |  |  |
|  | Nový Jubilejni  (neue Jubiläumsquelle) |  | 7,1 |  |  |  |
| **Lázně Hodonín** | Josefov BVJ ~ 1 Podluží |  | 50,6 mg/l bzw. 47 – 56 mg/l |  | https://www.laznehodonin.cz/de/lecba/prirodni-lecivy-zdroj | Bäder, Inhalation  Aufgrund des hohen Jodgehalts wird das Wasser 1:1 mit warmem Wasser verdünnt. |
|  | Josefov BVJ ~ 2 Prušánka |  |  |  |  |  |
|  | Josefov BVJ ~ 3 Josefka |  |  |  |  |  |
|  | Kontrollbohrung |  |  |  |  |  |
| **Lázně Lednice** | Brunnen in Charvatská Nová Ves | I-Br-Wasser | > 30 |  | https://www.lednicelazne.cz/de/stranka/informace/1/o-laznich/ | Bäder, Inhalation |
| **Lázně Darkov** |  | I-Br-Wasser | 25-45 |  | https://www.laznedarkov.cz/leciva-voda-solanka | Bäder, Wickel |
| **Lázně Klimkovice** |  | I-Br-Wasser | 40-50 |  | https://www.sanatoria-klimkovice.cz/cz/prirodni-lecivy-zdroj/jodobromova-solanka/ | Bäder, Wickel |

| **Italien** |  |  |  |  |  |  |
| --- | --- | --- | --- | --- | --- | --- |
| **Terme Stufe di Nerone – Bacoili** |  | Hyperthermales Na-Br-I-Wasser | 3 |  | https://www.termestufedinerone.it/it/acque-termali.html | Bäder, Inhalation, Schlamm |
| **Terme di Monticelli** |  | Na-Br-I-Wasser | 40,8 |  | https://www.termedimonticelli.it/acque.php | Bäder, Inhalation, Schlamm |
|  |  | S-Wasser | - |  |  |  |
| **Terme di Salsomaggiore** |  | Na-Br-I-Wasser | 61 |  | https://www.termedisalsomaggiore.it/it-IT/analisi-acqua-di-salsomaggiore.aspx | Bäder, Inhalation, Schlamm, orale Balneotherapie |
|  |  |  | 54 |  |  |  |
|  |  |  |  |  |  | "Acqua madre": konzentriertes Thermalwasser von 1000 l Brom-Jod-Wasser auf 50 l Acqua madre |
| **Riccione Terme** | Fonte Isabella | S-Na-Br-I-Mg-Wasser | 7,2 |  | https://www.riccioneterme.it/le-acque/ | Bäder, Inhalation, Schlamm |
|  | Fonte Claudia-Riccione |  | 1,3 |  |  |  |
|  | Fonte Celestine | leicht S-haltiges Na-Br-I-Mg-Wasser | 1,4 |  |  |  |
|  | Fonte Adriana | hochmineralisiertes Mg-S-Na-Br-I-Wasser | 1,9 |  |  |  |
| **Abano Terme & Montegrotto Terme** |  | hyperthermales Na-I-Br-Wasser | 0,82 |  | https://www.visitabanomontegrotto.com/de/thermen-de/elemente/thermalwasser-abano-montegrotto-terme/ | Thermalbad, Bäder, Inhalation, (Schlamm) |
| **Battaglia Terme (**Stabilimento Termale La Contea) |  | Na-Br-I-Wasser | 0,28 |  | https://www.termelacontea.com/de/ueber-uns/thermalwasser-eigenschaften | Thermalbad, Inhalationen, (Schlamm) |
| **Terme di Spezzano Albanese** | Fonte Thurio | Na-Br-I-Wasser | k. A. | k. A. | https://tdsface.wixsite.com/terme-di-spezzano/terme | Inhalationen, Schlamm |
|  | Acqua delle Grazie | hypotonisches NaCl-Wasser | k. A. | k. A. |  |  |
| **Terme Vico Equense – Scrajo Hotel** |  | Br-I-Na-S-Wasser | k. A. | k. A. | http://www.scrajoterme.it/centreo-termale-scrajo-penisola-sorrentina/ | Bäder, Inhalationen, Schlamm, Nasendusche |
| **Terme Brisighella** | Quelle Colombarino | S-Wasser | k. A. | k. A. | http://www.termedibrisighella.it/ | Bäder, Inhalationen, Schlamm |
|  | Quelle Igea Val D´Amone | Na-I-Wasser | k. A. | k. A. |  |  |
| **Terme Cervia** |  | Na-Br-I-Wasser  Inhaltsstoffe: Na, Ca, Cl, Mg, K, B, Li, Se, Br, I | k. A. | k. A. | https://www.terme.org/it/cure/indicazioni-terapeutiche/ | Bäder, Inhalationen, Schlamm |
| **Thermae Oasis**  (Lido delle Nazioni) | Quelle Ca´Rosa | Na-Br-I-Wasser | k. A. | k. A. | https://www.thermaeoasis.com/it-it/cure-termali | Bäder, Inhalationen, Schlamm, Nasendusche |
| **Terme della Fratta** |  | Na-I-Br-Wasser und  S-Wasser | k. A. | k. A. | https://www.termedellafratta.com/it/terme-e-salute/ayque-termali-salsobromoiodiche | Bäder, Inhalationen |
| **Terme di Castel San Pietro** |  | Na-Br-I-Wasser und  S-Wasser | k. A. | k. A. | https://termedicastelsanpietro.it/le-terme/acque-termali/ | Bäder, Inhalationen, Schlamm |
| **Terme di Castrocaro** |  | Na-Br-I-Wasser und  S-Wasser | k. A. | k. A. | https://www.termedicastrocaro.it/ | Bäder, Inhalationen, Schlamm, Trinkkur |
| **Terme di Riolo** | Acqua Margherita | Na-Br-I-Wasser | k. A. | k. A. | https://www.termediriolo.it/terme-riolo/acque/ | Bäder, Inhalationen, Schlamm, Trinkkur |
|  | Acqua Breta | HCO_3_-alkalisch-erdiges S-Wasser | k. A. | k. A. |  |  |
|  | Acqua Vittoria | Na-Br-I-Wasser | k. A. | k. A. |  |  |
| **Terme di Porretta** |  | Na-Br-I-Wasser und  S-Wasser | k. A. | k. A. | https://www.termediporretta.it/cure-termali/trattamenti-salsobromoiodiche/ | Bäder, Inhalationen, Schlamm, Spülungen |
| **Terme di Sant´Andrea** |  | HCO_3_-Alkaliwasser,  Na-Br-I-Wasser,  Cl-Na-Wasser,  Fe-K-Wasser und  S-Ca-Wasser | k. A. | k. A. | http://www.santandreabagni.it/le_terme.html | Bäder, Inhalationen, Trinkkur |
| **Terme della Salvarola** |  | Na-Br-I-Wasser,  S-Mg-HCO_3_-Wasser und  S-Wasser | k. A. | k. A. | https://www.termesalvarola.it/acqua-salso-bromo-iodica-a44 | Bäder, Inhalationen, Schlamm |
| **Terme di Punta Marina** |  | Na-Br-I-Ca-Mg-Wasser und  S-Wasser | k. A. | k. A. | https://www.termepuntamarina.it/acqua-termale/ | Bäder, Inhalationen |
| **Riminiterme** |  | Na-Br-I-Ca-Mg-Wasser | k. A. | k. A. | https://www.riminiturismo.it/visitatori/divertimento-e-relax/terme-e-benessere/centri-e-istiuti-termali/riminiterme-spa | Bäder, Inhalationen, Schlamm |
| **Terme di Stigliano** |  | S-Na-I-Wasser und  HCO_3_-SO_4_-Ca-Wasser | k. A. | k. A. | https://www.termedistigliano.it/it/cure-termali/ | Bäder, Inhalationen, Schlamm |
| **Terme di Sirmione** |  | S-Na-Br-I-Wasser | k. A. | k. A. | https://www.termedisirmione.com/it/salute/cura | Bäder, Inhalationen, Schlamm |
| **Salice Terme** | Quelle Fonte Sales | Na-Br-I-Wasser | k. A. | k. A. | https://termedisalice.it/cure-termali/ | Bäder, Inhalationen, Schlamm |
|  | Quelle Mont´Alfeo | S-Wasser | k. A. | k. A. |  |  |
| **Terme di Miradolo** |  | Na-Br-I-Li-Mg-S-Wasser | k. A. | k. A. | http://www.termedimiradolo.it/le-cure-convenzionate/ | Bäder, Inhalationen, Schlamm, Trinkkur |
| **Terme di Rivanazzano** |  | Na-Br-I-Wasser und  S-Wasser | k. A. | k. A. | https://www.termedirivanazzano.it/chi-siamo.html#acque | Bäder, Inhalationen, Schlamm |
| **Terme di Carignano** | Quelle Beatrice | S-Br-I-Wasser | k. A. | k. A. | http://www.termecarignano.it/home.html | Inhalationen, Nasendusche |
| **Monte Grimano Terme** |  | Alkalisches Wasser,  Na-Br-I-Wasser und  S-Wasser | k. A. | k. A. | http://www.montegrimano-terme.it/le-terme-di-montegrimano/ | Bäder, Schlamm |
| **Terme Santa Lucia** (Tolentino) |  | N-Br-I-Wasser,  S-Wasser und  HCO_3_-Wasser | k. A. | k. A. | https://www.termesantalucia.it/le-acque/ | Bäder, Inhalationen |
| **Acqui Terme** |  | Na-Br-I-S-Wasser | k. A. | k. A. | http://www.lagodellesorgenti.it/wp-content/uploads/2020/03/GH-Brochure-ITA-Welcome.pdf | Bäder, Inhalationen, Schlamm |
| **Terme Margherita di Savoia** |  | Na-Br-J-Wasser | k. A. | k. A. | http://www.termemargherita.it/de/unsere-kuranwendungen | Bäder, Inhalationen, Schlamm |
| **Terme Marino**  (Ali Terme) |  | S-Na-Br-I-B-Li-Wasser, alkalisch | k. A. | k. A. | http://www.termemarino.it/site/le-terme/ | Bäder, Inhalationen, Schlamm |
| **Terme di Sciacca** |  | S-Wasser und  Na-I-Br-Wasser | k. A. | k. A. | https://www.termesciaccaspa.it/piscine-molinelli | Becken: Piscine Molinelli |
| **Terme Termini Imerese** |  | Na-I-Br-Wasser | k. A. | k. A. | https://www.aquathermae.net/sizilien/die-thermen-von-termini-imerese/ | Bäder, Inhalationen, Schlamm |
| **Terme di Benetutti**  (Terme Aurora) |  | Na-Br-I-S-Wasser | k. A. | k. A. | https://www.termeaurora.it/trattamenti.html | Bäder, Inhalationen, Schlamm |
| **Terme di Casteldoria** |  | Na-Br-I-Wasser | k. A. | k. A. | https://termecasteldoria.it/ | Bäder, Inhalationen, Schlamm |
| **Terme di Galzignano** |  | Na-Br-I-Wasser | k. A. | k. A. | https://www.galzignano.it/de-DE/thermen | Bäder, Schlamm |
| **Terme della Versilia**  (Montignoso) |  | Na-Br-I-Wasser | k. A. | k. A. | http://www.termedellaversilia.com/terme.asp?idlink=11&lingua=_ita | Bäder |
| **Terme di Firenze Impruneta** | Fonte Antica | Na-Br-I-S-Wasser | k. A. | k. A. | https://www.aquathermae.net/toscana/terme-di-firenze/ | Bäder, Inhalationen |
|  | Fonte Celeste |  | k. A. | k. A. |  |  |

| **Schweiz** |  |  |  |  |  |  |
| --- | --- | --- | --- | --- | --- | --- |
| **Les Bains de Lavey** | Thermalquelle von Lavey | Na-SO_4_-Cl-S-Therme  enthält Li, F und B | 0,577 |  | https://www.tethyswater.ch/tethys-wasser/wasseranalyse/  https://www.bains-lavey.ch/de/das-thermalbad/ | Thermalbad, Mineralwasser |
| **Alpamare** |  | I-Sole-Becken | k.A. | k. A. | diverse Websites |  |
| **Splash e spa** |  | I-Sole-Becken | k.A. | k. A. | https://www.splashespa.ch/de/spa/jod-solebecken/ |  |

| **Slowakei** |  |  |  |  |  |  |
| --- | --- | --- | --- | --- | --- | --- |
| **Prírodné jódové kúpele Číž** | Themis-Quelle | I-Br-Wasser | k. A. |  | http://www.kupeleciz.sk/-historia | Bäder |
|  | Hygiea-Quelle |  | 26,9 |  | https://www.vodnesvety.sk/kupele-ciz/280-procedury-sluby | Trinkkur |
|  | Neptun-Quelle |  | k. A. |  |  | Bäder |
| **Bardejovské Kúpele** | Quelle Herkules | HCO_3_-Cl, Na, Fe-Carbonat, B - Wasser | 0,59 |  | https://www.slowakei-info.ch/archiv/de/tourismus/themen/tn-130612-1.html |  |
|  | Quelle Hlavný |  | - |  | https://www.kupele-bj.sk/liecba/prirodna-lieciva-voda | Trinkkur, Inhalationen, Bäder |
|  | Quelle Lekársky |  | - |  |  |  |
|  | Quelle Alžbeta |  | - |  |  |  |
|  | Quelle Klára |  | - |  |  |  |
|  | Quelle Napoleon |  | - |  |  |  |
|  | Quelle Anna |  | - |  |  |  |
|  | Quelle Kolonádny |  | - |  |  |  |
| **Kúpele Nimnica** | Natürliche Heilquelle B7 | HCO_3_-Na-I-Wasser mit erhöhtem H-Gehalt | 1,33 |  | http://www.kupelenimnica.sk/sk/stranka/liecba | Bäder, Inhalation |
| **Termálne kúpalisko Podhájska** | Podhájska-Quelle |  | 3,23 |  | http://www.tkpodhajska.sk/kupalisko/geotermalny-pramen | Thermalbad |
| **Termálne kúpaliska vincov les, s.r.o.** |  |  | 0,56 |  | https://www.vincovles.com/termalne/ | Thermalbad |

| **Slowenien** |  |  |  |  |  |  |
| --- | --- | --- | --- | --- | --- | --- |
| **Thalasso Strunjan** | Meerwasser |  | < 50 |  | https://www.terme-krka.com/de/de/strunjan/spa/thalassotherapie/ | Therme mit Meerwasser |
| **Therme Portorož** |  |  | k. A. | k. A. |  |  |
